# Transcriptome and Metabolome Analyses of Glucosinolates in Two Broccoli Cultivars Following Jasmonate Treatment for the Induction of Glucosinolate Defense to *Trichoplusia ni* (Hübner)

**DOI:** 10.3390/ijms17071135

**Published:** 2016-07-15

**Authors:** Kang-Mo Ku, Talon M. Becker, John A. Juvik

**Affiliations:** 1 Division of Plant and Soil Sciences, West Virginia University, Morgantown, WV 26505, USA; kangmo.ku@mail.wvu.edu; 2 Department of Crop Sciences, University of Illinois at Urbana–Champaign, Urbana, IL 61801, USA; tbecker2@illinois.edu

**Keywords:** insect resistance, jasmonic acid, glucosinolate

## Abstract

Lepidopteran larvae growth is influenced by host plant glucosinolate (GS) concentrations, which are, in turn, influenced by the phytohormone jasmonate (JA). In order to elucidate insect resistance biomarkers to lepidopteran pests, transcriptome and metabolome analyses following JA treatments were conducted with two broccoli cultivars, Green Magic and VI-158, which have differentially induced indole GSs, neoglucobrassicin and glucobrassicin, respectively. To test these two inducible GSs on growth of cabbage looper (*Trichoplusia ni*), eight neonate cabbage looper larvae were placed onto each of three plants per JA treatments (0, 100, 200, 400 µM) three days after treatment. After five days of feeding, weight of larvae and their survival rate was found to decrease with increasing JA concentrations in both broccoli cultivars. JA-inducible GSs were measured by high performance liquid chromatography. Neoglucobrassicin in Green Magic and glucobrassicin in VI-158 leaves were increased in a dose-dependent manner. One or both of these glucosinolates and/or their hydrolysis products showed significant inverse correlations with larval weight and survival (five days after treatment) while being positively correlated with the number of days to pupation. This implies that these two JA-inducible glucosinolates can influence the growth and survival of cabbage looper larvae. Transcriptome profiling supported the observed changes in glucosinolate and their hydrolysis product concentrations following JA treatments. Several genes related to GS metabolism differentiate the two broccoli cultivars in their pattern of transcriptional response to JA treatments. Indicative of the corresponding change in indole GS concentrations, transcripts of the transcription factor *MYB122*, core structure biosynthesis genes (*CYP79B2*, *UGT74B1*, *SUR1*, *SOT16*, *SOT17*, and *SOT18*), an indole glucosinolate side chain modification gene (*IGMT1*), and several glucosinolate hydrolysis genes (*TGG1*, *TGG2*, and *ESM1*) were significantly increased in Green Magic (statistically significant in most cases at 400 µM) while *UGT74B1* and *MYB122* were significantly increased in VI-158. Therefore, these metabolite and transcript biomarker results indicate that transcriptome profiling can identify genes associated with the formation of two different indole GS and their hydrolysis products. Therefore, these metabolite and transcript biomarkers could be useful in an effective marker-assisted breeding strategy for resistance to generalist lepidopteran pests in broccoli and potentially other *Brassica* vegetables.

## 1. Introduction

Broccoli (*Brassica oleracea* ssp. *italica*) is a frequently consumed vegetable in the United States and in other countries. It contains potential health promoting bioactive compounds including glucosinolates and dietary antioxidants, such as carotenoids, tocopherols, and flavonoids [1,2], particularly quercetin and kaempferol [3], which have been reported as potential anticancer agents [4]. Recent publications suggest that exogenous application of methyl jasmonate (MeJA) enhances cancer chemopreventive and/or antioxidant activity of broccoli and other *Brassica* crops [5,6,7,8]. Thus, treatment with jasmonic acid (JA) and its derivatives (hereafter JAs) can be a useful agricultural application to improve phytonutrient quality of broccoli and other *Brassica* crops.

JAs are important compounds in plant defense. In response to JA or MeJA treatment, concentrations of several different direct or indirect defense compounds, including proteinase inhibitors, polyphenol oxidases, nicotine, and glucosinolates, have been shown to increase [9,10,11,12]. Several studies have reported that herbivore behavior or development is affected by jasmonate elicitation [13]. For example, JA treatments have been shown to decrease the abundance of caterpillars, flea beetles, aphids, and thrips in tomatoes under field conditions [14]. JA treatments on Brussels sprouts changed oviposition preference of *Pieris rapae* and *Pieris brassicae* and the time from egg hatch to pupation [15]. In particular, there have been a number of studies investigating the effect of glucosinolates on insect herbivory [16].

In *Brassica* plants, JAs, plant signal transduction compounds associated with herbivore defense, can act as elicitors to enhance glucosinolate (GS) biosynthesis, which has been reported as a defense mechanism against both phloem-feeding and chewing insect herbivores [17]. MeJA treatment typically increases indole GSs in *Brassica* crops [5,6,8,10,18,19,20]. Biosynthesis of indole GSs starts with conversion of the precursor amino acid, tryptophan, to the corresponding aldoxime by the cytochrome P450 monooxygenases, *CYP79B2* or *CYP79B3* [21,22]. The aldoxime enters the GS core biosynthesis pathway to form indol-3-ylmethyl-desulfo-GS and is then sulfated by the sulfotransferase *SOT16* to form the GS glucobrassicin [23]. Side chain modification of glucobrassicin by members of the cytochrome P450 family *CYP81F4* leads to formation of hydroxy-indol-3-ylmethyl GS, which can then be methylated to 1-methoxy-indol-3-ylmethyl GS (neoglucobrassicin) by a specific class of plant family 2-*O*-methyltransferases [24]. Side chain modification is a critical step in determining bioactivity of indole GS compounds because 4-methoxylation of glucobrassicin results in formation of the antifungal indole GS, 4-methoxy-indol-3-ylmethyl GS [25].

Previous studies have reported plant defense capabilities of indole GS hydrolysis products for a range of plant pests. Glucosinolates do not provide biological activity, rather it is the hydrolysis of GSs by the endogenous enzyme myrosinase that generates a range of GS hydrolysis products with bioactivity. 4-methoxy glucobrassicin and neoglucobrassicin hydrolysis products have been shown to have stronger deterrent effects than glucobrassicin hydrolysis products on aphids [26].

The products produced upon the hydrolysis of GSs differ depending on the activity of epithiospecifier protein (ESP) and the influence of the epithiospecifier modifier protein (ESM; gene for ESM: *ESM1*). Specifically, using the indole GS glucobrassicin, ESP activity was shown to decrease the formation of the isothiocyanate (ITC)-derived carbinol in favor of a nitrile hydrolysis product, while ESM activity was shown to act antagonistically against ESP activity [27]. Based on these results and due to the structural similarity of glucobrassicin and neoglucobrassicin, it is plausible that ESP influences neoglucobrassicin hydrolysis similarly by decreasing the formation of 1-methoxy indole-3-carbinol (MI3C) in favor of 1-methoxy indole-3-acetonitrile (MI3A), with ESM antagonizing that activity and influencing formation towards MI3C. Recent research has shown that MI3C has mutagenic or genotoxic effects in mammalian and bacterial cell studies [28,29]. Thus, *Brassica* crops with high neoglucobrassicin concentrations and low ESP and/or high ESM activity could act specifically as a deterrent or antibiotic against insects or other organisms.

Lepidopteran species are some of the most serious insect pests to *Brassica* vegetables. Additionally, larvae of many lepidopteran insect pests typically stay on the host plant on which they hatched due to limited dispersal capacity [15,30]. For these reasons, there has been interested in how jasmonate treatments applied to host plants affect the development of lepidopteran larvae. Previously, our studies have reported that indole glucosinolates in broccoli florets were significantly induced by exogenous MeJA sprays [6,19]. The florets of most broccoli genotypes increased in neoglucobrassicin concentrations in response to MeJA treatments, but the florets of a few broccoli cultivars increased both glucobrassicin and neoglucobrassicin, or glucobrassicin predominantly. The cultivar, Green Magic, has shown high amplification of neoglucobrassicin concentrations in floret tissue following MeJA treatment [6,19]. In contrast, the doubled-haploid genotype, VI-158 was observed to increase glucobrassicin content after MeJA treatment [20]. Due to the structural difference between glucobrassicin and neoglucobrassicin, we hypothesized that these two different broccoli cultivars may show differences in insect defense capabilities. However, the effect of different JA-inducible indole GSs in broccoli on the development of lepidopteran larvae is not fully understood. Although there have been several reports on the effect of GSs on insect herbivores with mutant *Arabidopsis* lines [16,31,32], research of neoglucobrassicin on insect herbivores is lacking because this indole GS tends to be absent or at a very low levels in *Arabidopsis* [33]. Previous research with broccoli suggests that the concentrations of indole GSs is mainly controlled by environmental effects (such as insect damage or JA treatment) rather than genetic effects [19,34]. Thus, we chose JA as elicitor to manipulate indole GSs because previous studies found that GS concentrations are the main anti-insect herbivore factor from mutant *Arabidopsis* line experiments [16,32,33]. In this experiment, we selectively increased either neoglucobrassicin (Green Magic) or glucobrassicin (VI-158) with JA treatments. As a result, we were able to test anti-herbivore effect of these two inducible indole GS on cabbage looper growth and survival. JA treatment is a reproducible and convincing tool to mimic the insect damage so that we can induce not only indole GS biosynthesis but also induce other defense related mechanisms including myrosinase, ESP, and ESM enzymes. Thus, our indole GS manipulation with JA might have potential confounding effects in comparing the anti-herbivore effect of two different indole GSs. However, inducing overall defense mechanisms with JA treatment allows us to evaluate comprehensive insect defense mechanisms including inducible indole GS, GS hydrolysis, and other mechanisms that are potentially of value in a breeding program to improve insect resistance. The first objective of this research was to compare the anti-herbivore effect of two broccoli cultivars (Green Magic and VI-158) with differing JA-induced GS profiles to cabbage looper (*Trichoplusia ni*). Another objective of this research was to establish resistance biomarkers for lepidopteran pests from targeted GS transcriptome and metabolome studies to be used as possible lepidopteran resistance selection tools in *Brassica* breeding programs.

## 2. Results

### 2.1. Effect of JA (Jasmonate) Treated Broccoli on Growth and Survival of Cabbage Looper

**Figure 1:**
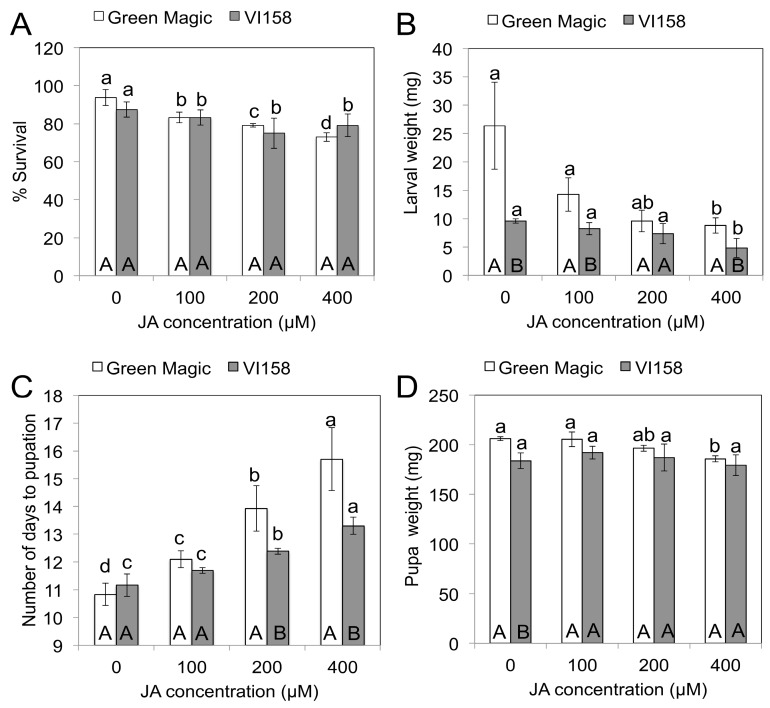
The effect of jasmonate on % survival (**A**); larval weight (**B**); number of days to pupation (**C**); and pupal weight (**D**) of cabbage looper (*Trichoplusia ni*) after 5 days feeding on two different broccoli cultivars. Different lower case letters above error bar indicate significant differences between treatments within a cultivar, while upper case letters inside bar indicate differences between cultivars within treatments as determined by Fisher’s LSD test at *p* = 0.05. The results are presented as means ± SD (*n* = 3).

**Figure 2:**
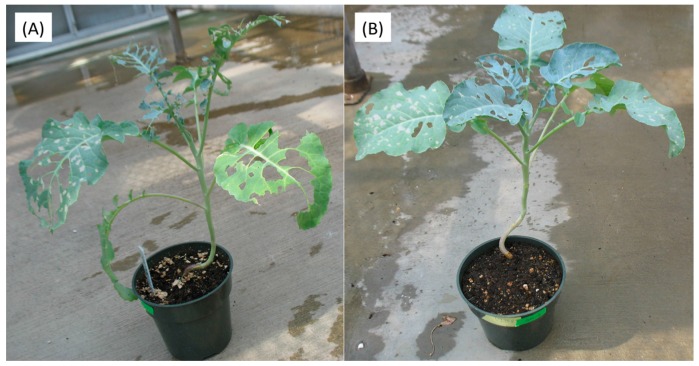
Representative plants after *Trichoplusia ni* feeding for control (**A**) and 400 µM JA treated VI-158 broccoli plants (**B**). The pictures were taken 10 days after larval infestation.

Three days after the JA treatments, insect feeding was initiated on plants of the two different broccoli cultivars. Five days after feeding initiation (DAFI), cabbage looper larval weights and survival were determined. Plants subjected to higher concentrations of JA treatments showed reduced cabbage looper survival (Figure 1A). There were no significant survival differences between larvae fed on Green Magic and VI-158 for any of the JA treatments. Larval weights measured on the fifth DAFI declined with increasing JA concentrations in both cultivars (Figure 1B). The weight reducing effect of the JA treatments may be due to a feeding deterrence observed in this experiment (Figure 2). This figure shows 400 µM JA treated VI-158 broccoli plants displaying reduced feeding damage compared to control plants after five days of feeding. This was also observed in the Green Magic cultivar (but pictures were not taken). Larval weight reduction induced by increasing concentrations of JA treatments was more pronounced in the Green Magic genotype; except for the 200 µM JA treatment, Green Magic-fed cabbage loopers had significantly higher average larval weight than VI-158-fed cabbage loopers. This was mainly due to the observation that larvae feeding on control Green Magic plant tissue gained more weight compared to control VI-158-fed cabbage loopers (Figure 1B). These results may partially be associated with the higher concentrations of protein in Green Magic tissue (Figure S1). The protein concentration of Green Magic seedlings was about 3-fold higher than VI-158 in the 0 and 100 µM JA treatment groups and at least 1.5-fold higher than VI-158 in the 200 and 400 µM JA treatment groups. It has been previously reported that herbivorous insects respond to lower protein levels with reduced growth [35], or alternatively, increased larval growth rates were observed when diets were supplemented with additional protein [36]. Although our results show that Green Magic broccoli leaves have identical levels of protein at 0 and 100 µM JA treatment groups, the larval weight was numerically decreased as JA concentration increased from 0 to 100 µM. This implies the larval weight change was not solely by protein level change.

### 2.2. JA Effect on Trichoplusia ni Pupation and Pupal Weight

After the replacement of dead larvae from the initial five-day feeding trial, all larvae survived until pupation, except in the 400 µM JA treatment group in Green Magic (23/24 survived). However, in the initial 5-day feeding experiment, >20% of the larvae died when fed with 400 µM JA treated plants of either variety (Figure 1A). This indicates that JA-inducible compounds are more toxic to early larval instars. It has been previously reported that in lepidopteran larvae, mortality spikes during the first instar and then levels off as the larvae grow and become better able to detoxify and/or excrete the plant’s toxins [37]. The average number of days to pupation of cabbage loopers was delayed by JA treatment in both broccoli cultivars; the caterpillars on the 400 µM JA treated plants pupated on average after 15.7 and 13.3 days compared to 10.8 and 11.2 days in control plants of Green Magic and VI-158, respectively (Figure 1C). There was a significant pupation delay in Green Magic-fed cabbage loopers in the 100, 200 and 400 µM JA treatments and in the 200 and 400 µM JA treatments for VI-158-fed larvae. Green Magic-fed cabbage loopers had significantly delayed pupation compared to VI-158-fed larvae at 200 and 400 µM JA treatments. Delayed pupation has been reported in a previous study with larvae of *Pieris brassicae* and *Pieris rapae* fed with JA-treated Brussels sprouts tissue [15]. Pupal weight was not significantly influenced by JA-treated VI-158 broccoli, but pupal weight for larvae reared on JA-treated Green Magic tended to decrease with increasing JA concentration (Figure 1D). In particular, larvae feeding on the 400 µM JA-treated Green Magic plants showed significantly decreased pupal weight, compared to the control. JA treatment significantly increased neoglucobrassicin in Green Magic up to 15-fold over the control while glucobrassicin was significantly increased in VI-158 up to 7-fold over the control (Table 1). In addition to changes in indole GSs, the glucoiberin concentration was significantly increased by JA treatment in Green Magic. Additionally, the gluconasturtiin concentration in VI-158 was significantly decreased by JA treatment (Table 1). While indole GSs accounted for 59% of total GSs in control Green Magic tissue, they contributed 88% of total GSs in the 400 µM JA-treated plants. Indole GSs accounted for 65% of total GSs in control VI-158 broccoli plants but accounted for 98% of total GSs in 400 µM JA treated plants. In VI-158 broccoli plant tissue, the JA treatment primarily increased glucobrassicin. This suggests that the reduced larval weight and delayed pupation for VI-158-fed larvae may be due to the increase in glucobrassicin concentrations. In Green Magic broccoli tissue, JA treatment had a significant influence on survival, larval and pupal weight, and number of days to pupation, often with a significant dosage response. In Green Magic, neoglucobrassicin concentrations were most responsive to increasing JA concentrations, with a moderate increase also observed for glucoiberin. This suggests that for Green Magic-fed larvae, the JA-mediated increase in neoglucobrassicin concentrations may account for differences in larval survival and development, with perhaps some effect also attributed to increases in glucoiberin.

MI3C and 1-methoxyindole-3-carboxaldehyde (MI3CA) were the major hydrolysis products in Green Magic while indole-3-carboxaldehyde (I3CA), MI3C, and MI3CA were the primary hydrolysis products in VI-158 (Table 2). In general, Green Magic broccoli plants had higher hydrolysis product concentrations for both aliphatic and indole GSs compared to VI-158, with the exception of the glucobrassicin hydrolysis products, I3C and I3CA. VI-158 did not display elevated concentrations of I3C or I3CA following JA treatments despite showing a large increase in glucobrassicin, their precursor GS. This may be related to the instability of indole-3-carbinol in solution and the number of I3C derivatives that are produced, many spontaneously, in vivo [32]. Previous research has reported that JA-treated kale showed significantly lower indole-3-carbinol concentrations than its precursor GS, glucobrassicin [5]. In our previous study, we detected diindolylmethane that is a dimer of I3C.

### 2.3. Gene Expression Analysis

**Figure 3:**
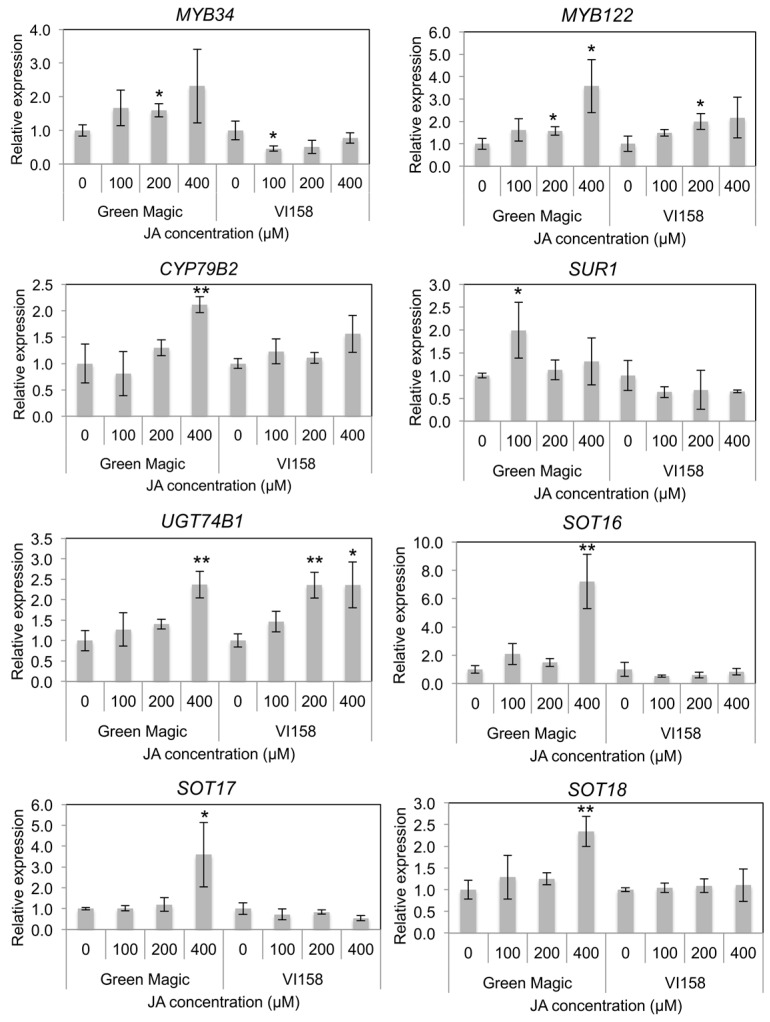
Histograms representing changes in relative transcript abundance of transcription factors and glucosinolate core structure synthesis genes for different cultivar/treatment combinations. Asterisks indicate significant differences from the control within the same cultivar determined by Student’s *t*-test at *p* = 0.05 (*) and *p* = 0.01 (**), respectively.

Indole GS biosynthesis and hydrolysis gene expression was measured by quantitative Real Time- Polymerase Chain Reaction (qRT-PCR) in order to determine possible reasons for differences in glucosinolate inducibility from JA treatments. A previous study reported that transcriptional factors including *MYB34* and *MYB122* regulate JA-mediated indole GS biosynthesis in *Arabidopsis* [38]. Gene expression of both *MYB34* and *MYB122* increased with increasing JA concentrations in Green Magic seedlings while only *MYB122* showed this response in VI-158 (Figure 3 and Figure S2). The gene expression of *MYB34* in VI-158 decreased compared to the control for all JA treatments. The gene expression of the indole core biosynthesis genes, including *CYP79B2*, *UGT74B1*, and *SOT16*, were generally only increased in Green Magic and only significantly at the 400 µM JA treatment level. In addition, aliphatic GS-associated sulfotransferases, *SOT17* and *SOT18*, showed significant increases in transcription with 400 µM JA treatments. This result may explain to the increase in glucoiberin observed with increasing JA concentrations. It should be noted, however, that although the 400 µM JA treatment significantly increased transcription of all measured sulfotransferases, the increase was greatest for *SOT16* (>6-fold; Figure 3 and Figure S2), the sulfotransferase related to indole GS biosynthesis. For VI-158, transcription of *UGT74B1* was significantly increased with both 200 and 400 µM JA treatments, while this was true for only the 400 µM treatment in Green Magic. Somewhat contrary to the observed pattern, *SUR1* transcription was significantly increased by JA treatments only in Green Magic, but with the only notable increase (≈2-fold) at the 100 µM JA treatment level.

**Figure 4:**
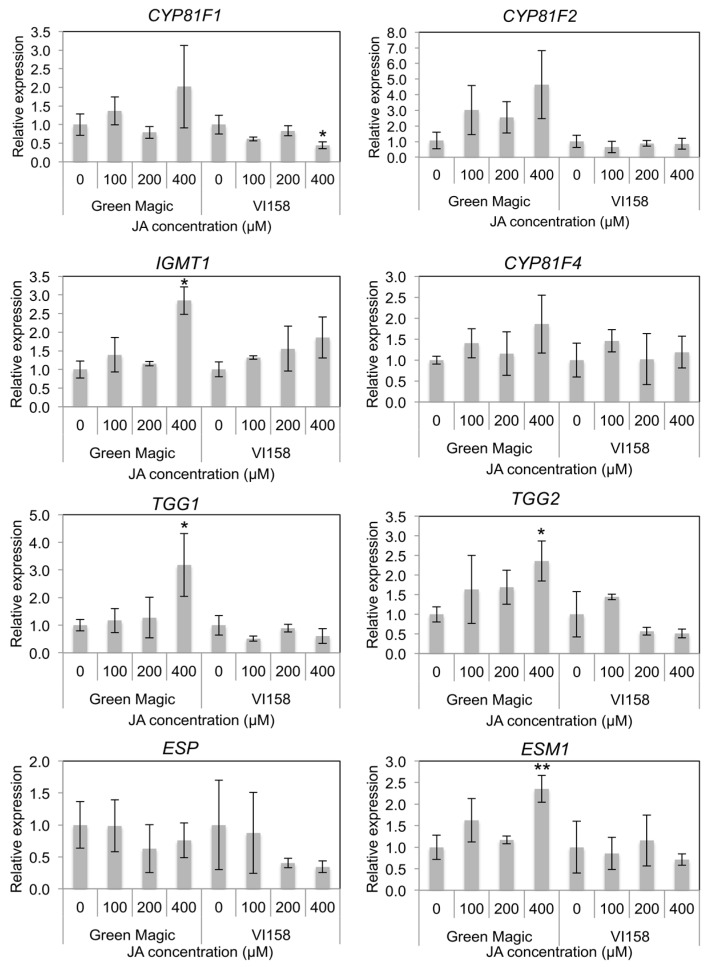
Histograms representing changes in relative transcript abundance of glucosinolate side chain modification, and hydrolysis related genes for different cultivar/treatment combinations. Asterisks indicate significant differences from the control within the same cultivar determined by Student’s *t*-test at *p* = 0.05 (*) and *p* = 0.01 (**), respectively.

In indole GS side chain modification-related gene expression, *CYP81F1*, *CYP81F2*, and *CYP81F4* were not significantly changed after JA treatment in either cultivar (Figure 4 and Figure S2). However, some trends were observable in the transcription of these genes that differed between the two cultivars. In general, Green Magic showed an increase in transcription of *CYP81F* family genes with increasing JA concentration, while no increase, or even a slight decrease, was observed in VI-158 (Figure 4 and Figure S2). The gene expression of *IGMT1*, which is responsible for O-methyltransferase activity needed to create neoglucobrassicin and 4-methoxy glucobrassicin, was significantly increased only in the 400 µM JA treated Green Magic broccoli plants (Figure 4 and Figure S2). The gene expression of myrosinase enzymes *TGG1* and *TGG2* was also only increased in 400 µM JA-treated Green Magic broccoli tissue. The gene expression of *ESM1* was significantly increased in 400 µM JA-treated Green Magic broccoli seedlings although the gene expression of *ESP* was not significantly changed by JA treatment. These increases in *TGG1*, *TGG2*, and *ESM1* transcription can help to explain the stark increase in MI3C concentrations in Green Magic. It is known that *ESM1* antagonizes *ESP* activity and influences GS hydrolysis towards isothiocyanates for a number of GSs. In the case of neoglucobrassicin hydrolysis, the isothiocyanate formed is highly unstable and is known to rearrange into MI3C and NeoASG, among other products [39]. Additionally, increasing the abundance of myrosinase may lead to the formation of more isothiocyanates, as this is the major hydrolysis product produced in the absence of specifier proteins and with limiting Fe^2+^ [40,41].

## 3. Discussion

Previous work done using *Arabidopsis thaliana* plants with mutations that greatly reduced levels of aliphatic GSs, indole GSs, or both, demonstrated that the growth of *T. ni*, *Manduca sexta*, and *Spodoptera exigua* larvae, three lepidopteran species, were all negatively affected by the presence of aliphatic GSs [31]. Only growth of *S. exigua* larvae was negatively influenced by presence of indole GSs [31]. Transcription factors *MYB28* and *MYB29* are partially redundant, but double knock-out mutants do not produce aliphatic GSs. The herbivory damage of these double knock-out plants correlated inversely to the levels of aliphatic GSs observed in those plants fed on by the generalist lepidopteran pest, *Mamestra brassicae* [16]. Similar results were seen in another study, which concluded that aliphatic GSs had growth inhibitory effects only on *Mamestra brassicae*, and not the specialist *Pieris rapae,* reared on feral *Brassica oleracea* [42]. These studies indicate the importance of aliphatic GSs for defense from insect herbivores, particularly generalists like *Mamestra brassicae* and *T. ni*. In particular, the result from Müller et al. [31] showing the effect of aliphatic GSs on *T. ni* supports the hypothesis that the significantly larger reduction in *T. ni* survival and development with increasing JA concentration for Green Magic-fed larvae compared to VI-158-fed larvae seen in this experiment can be at least partially attributed to increases in glucoiberin (Table 1). The decreasing protein levels observed in the higher JA concentration treatments of Green Magic plants could have added additional developmental restrictions (*r* = 0.840, *p* = 0.009, *n* = 8; Table S2). Previous metabolomics approach revealed the level of glucose, sucrose and amino acids showed a decrease after methyl jasmonate treatment on *Brassica rapa* [18]. It is possible that high accumulation of GSs may lead to a limitation of primary metabolites for the cabbage lopper. It is difficult to assess the effect of neoglucobrassicin on insect herbivores because this indole GS tends to be at a very low level in *Arabidopsis* [33], even after insect damage [17]. So far, there is only limited information on how indole GSs influence herbivore behavior and development. A previous study reported that growth and development of *Pieris rapae* was negatively correlated with neoglucobrassicin concentrations in three wild cabbage populations [43]. Another publication from Harvey and his colleagues reported that multivariate statistics revealed that pupal mass and development time of *Pieris brassicae* correlated with foliar GS chemistry, wherein levels of neoglucobrassicin appeared to be the most important factor [44]. Kim and Jander [26] reported that the hydrolysis products from neoglucobrassicin and 4-methoxy glucobrassicin had higher antifeedant effects than glucobrassicin hydrolysis products on *Myzus persicae*. Similarly, a high concentration of neoglucobrassicin in JA-treated Green Magic may contribute to an increased antifeedant effect on cabbage looper herbivory as seen in this study (Table 1). However, to our knowledge there have been no investigations comparing the relative antifeedant activity between neoglucobrassicin and glucobrassicin for lepidopteran pests. In this experiment we were able to selectively increase either neoglucobrassicin or glucobrassicin in Green Magic or VI-158, respectively, by using JA as an elicitor (Table 2). It has been previously reported that MI3C is a mutagenic or toxic compound to mammalian cells [28,29]. In rat animal study, MI3C had more efficient inducer of cytochrome P-450 1A1 than I3C [45]. In a human cancer cell line study, MI3C inhibited cell growth of DLD-1 and HCT-116 human colon cancer cell lines [46]. Taken together, the 1-methoxylation of I3C to form MI3C may significantly change the toxicity of MI3C by increasing hydrophobicity and cell membrane penetration. The observed toxicity of this compound may also help to explain the superiority of Green Magic to VI-158 in terms of insect growth/survival reduction following JA treatments (Table 2, Figure 1a,b). The number of days to pupation was significantly correlated with MI3CA (*r* = 0.929, *p* = <0.0001, *n* = 8), MI3C (*r* = 0.927, *p* = <0.0001, *n* = 8), neoASG (*r* = 0.86, *p* = 0.006, *n* = 8), total indole GSs (*r* = 0.977, *p* = <0.0001, *n* = 8), and total GSs (*r* = 0.913, *p* = 0.002, *n* = 8) concentrations (Table S2). Green Magic showed a much larger increase in MI3C with JA treatment compared to VI-158, likely due to the significantly larger increase in neoglucobrassicin, the precursor GS (Table 1). Similarly, JA treated Brussels sprouts plants delayed the number of days from hatch to pupation of *Pieris rapae* [15]. In our experiment, the growth delay was possibly due to the antifeedent effect of inducible indole GS since the feeding damage of MeJA treated plants was less than in control groups (Figure 3). However, the complex genetic differences between these two broccoli cultivars make it difficult to determine a direct causal relationship between individual glucosinolate breakdown products and insect performance. Hence, more insect experiments are needed to confirm variation in antifeedant activity between hydrolysis products of two indole GS. Since the MI3C compound is not commercially available, so far only a few human cell line studies have been conducted with synthesized MI3C [45,46]. Due to the difficult synthesis process, previous bioassays with isolated neoglucobrassicin and exogenous myrosinase are limited [19,47,48]. It was not feasible to conduct artificial diet feeding tests in this study because this requires large amounts of neoglucobrassicin and the development of a process to generate encapsulated myrosinase [49] to avoid loss of GS hydrolysis products from direct contact between neoglucobrassicin and myrosinase prior to insect herbivory.

Although a number of significant transcriptional responses were seen in our study following JA treatment, many were not significant at lower JA concentrations (Figure 3, Figure 4 and Figure S2). Previous studies on broccoli and pak choi reported that expression of JA-responsive genes was significantly increased two days after JA treatment [6,50]. The present study used plant tissues harvested three days after JA treatment for transcript quantification. For this reason, gene transcript quantifications could be underestimated if their peak level is indeed two days after treatment [6]. This may also explain why the 400 µM JA treated plants still showed up-regulation in several genes while the plants from other treatments did not (Figure 3, Figure 4 and Figure S2). The largest differences in gene expression response to JA treatment between Green Magic and VI158 were observed in the transcriptional factor *MYB34*, core indole biosynthesis genes including *SOT16*-*SOT18*, the side chain modification gene *IGMT1*, myrosinase genes *TGG1* and *TGG2*, and the hydrolysis regulator *ESM1*. These observations of the transcriptional regulation imposed by JA treatments agrees well with the changes seen in glucosinolate and hydrolysis product profiles (Table 1 and Table 2). The JA treatment increased neoglucobrassicin and its hydrolysis products in Green Magic. In addition, elevated transcript abundance of *TGG1*, *TGG2*, and *ESM1* may contribute to generate more isothiocyanate or isothiocyanate-derived hydrolysis compounds more rapidly after initial tissue disruption (i.e., herbivory). Although rates of hydrolysis product production was not measured in this experiment, in a previous study MeJA treated broccoli floret samples showed significantly higher hydrolysis products than control samples in five different cultivars [19]. Increased indole GS concentrations with changed myrosinase and myrosinase-related gene expression directly effects levels of hydrolysis products. This may have been a factor in the superior antifeedant activity of Green Magic to VI-158 and should be further investigated in future work.

In addition, by spraying JA, we mimicked responses of insect defense-related genes and defense compounds in a scenario that would be experienced by these crops under field conditions. Experiments using mutants can significantly up- or down-regulate the gene expression of specific GS biosynthesis genes, but this method does not always generate the desired change in physiochemical profiles. Blocking aliphatic GS biosynthesis in *Arabidopsis* increased glucobrassicin concentrations in *myb28* or *myb29* knockout lines or in double mutant plants [16]. Blocking *CYP79F1* or *CYP79F2* genes using RNAi significantly changed various enzyme activities, abundance of amino acids, and various plant hormone concentrations, resulting in different phenotypes [51]. Studies using mutant plants or RNAi technology can provide insights, but this is not always a realistic situation. Alternatively, profiling of GSs and related genes’ expression modifications in response to stress elicitors can mimic more realistic changes in plant physiology, including expression of myrosinase, as well as *ESP*, *ESM1*, and other myrosinase-related cofactors.

**Figure 5:**
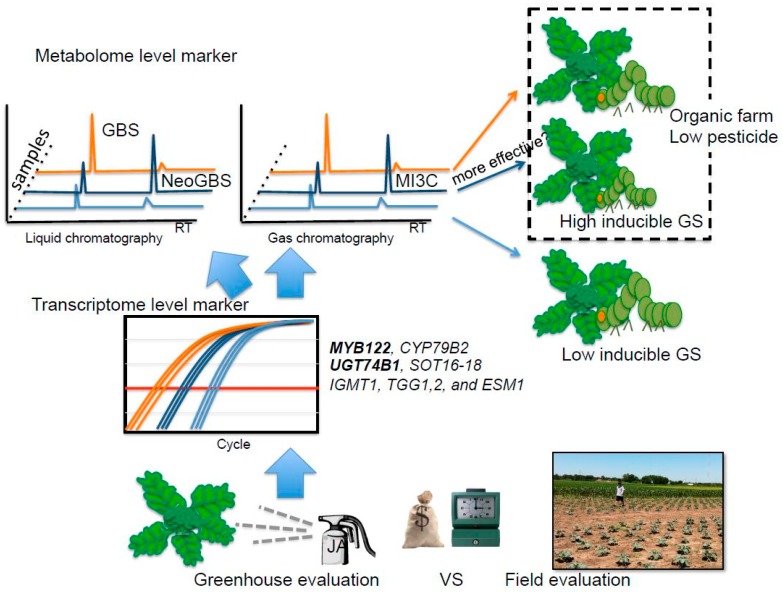
Possible breeding scheme for insect resistant germplasm by using JA/MeJA application to evaluate inducible GS and other defense mechanisms at the metabolite and transcriptome level. GBS: glucobrassicin, NeoGBS: neoglucobrassicin; MI3C: *N*-methoxyindole-3-carbinol. Transcript levels of *MYB122* and *UGT74B1* were commonly increased in two broccoli cultivars.

Taken together, the results obtained in this study suggest that both inducible glucobrassicin and neoglucobrassicin may provide better antifeedant activity in broccoli for generalist lepidopteran pests, specifically *T. ni*. However, this still remains to be confirmed (Figure 5). Previously, Ku et al. [5] showed, in a study using kale, that inducible indole GSs were significantly increased within 24 h in apical leaf tissue. Thus, metabolite-based insect resistance screening will not require the same level of resources compared to field evaluation (Figure 5). The tentative insect resistance compounds, MI3C and other hydrolysis products from neoglucobrassicin, were reliably detected by GC or GC-MS. Hence, these hydrolysis product metabolites can be good biomarkers. The Green Magic cultivar showed dramatic transcriptional response in indole GS biosynthesis genes and transcriptional regulatory genes to JA treatment. This cultivar also showed favorable changes in gene expression for hydrolysis-related genes (*ESP* and *ESM1*), resulting in the production of more potent insect defense compounds (isothiocyanate or isothiocyanate-derived GS hydrolysis products instead of nitrile GS hydrolysis products). Transcript levels of *MYB122* and *UGT74B1* were commonly increased in the two broccoli cultivars (Figure 3). Thus, transcriptional profiling of these genes can be utilized to screen for insect resistance phenotypes for broccoli breeding programs with the application of JA/MeJA treatments. As an added benefit to this screening strategy, breeding programs would be more likely to produce varieties that are more responsive to jasmonate elicitation (Figure 5). These jasmonate-responsive varieties would allow the plants to more quickly and strongly respond to natural jasmonate production when subjected to herbivory. Highly responsive cultivars may be appropriate in organic or low pesticide production systems (Figure 5). It may also allow for growers to use jasmonate sprays in a preventive strategy utilizing natural defense mechanisms to keep insect herbivory pressure to a minimum. Future research is needed to determine the feasibility of this strategy, as overstimulation of defensive mechanisms may lead to yield drag [52].

## 4. Materials and Methods

### 4.1. Broccoli Cultivation

The broccoli genotypes used in this experiment were VI-158, a doubled-haploid variety (courtesy of Mark W. Farnham, USDA, Charleston, SC, USA), and the F1 commercial hybrid “Green Magic” (Sakata Seed Co., Morgan Hill, CA, USA). Seeds of each broccoli variety were germinated in 32 cell plant plug trays filled with Sunshine^®^ LC1 (Sun Gro Horticulture, Vancouver, BC, Canada) professional soil mix. Plants were grown in a greenhouse at the University of Illinois at Champaign-Urbana under a 25/18 °C and 14/10 h: day/night temperature regime with HID lighting provided from 06:00 to 20:00 h if light intensities fell below 2670 μmol·m^−2^·s^−1^. Four weeks later, plants in the vegetative growth stage were transferred to 1-L pots in the greenhouse under the same conditions. Broccoli plants with 8 fully developed leaves were selected for the experiment.

### 4.2. Feeding of Trichoplusia ni (Hübner)

The development of first instar larvae to pupation was observed on control and JA-treated plants. Control plants were sprayed with a 0.1% Triton X-100 (Sigma-Aldrich, St. Louis, MO, USA) solution and JA-treated plants with a solution of 100, 200, and 400 µM JA with 0.1% Triton X-100. The treatment concentrations were determined from a previous study [7]. Twenty-four newly hatched cabbage looper larvae were evenly distributed (eight larvae/plant as one biological replication) using a paint brush on three plants per treatment, three days after JA treatments, and were placed in cages (66–46 cm, Collapsible hamper, Whitmor, Southaven, MS, USA) in a greenhouse at 25–27 °C and 50%–70% relative humidity. Five days after feeding initiation (DAFI), larvae weight and survival were determined from each of the broccoli plants. Also at 5 DAFI, dead larvae were replaced with larvae grown on the same treatments under the same conditions for the determination of time to pupation and pupal weight. Following pupation, pupae from different treatments were collected every day, and the number of pupae and their weights were recorded.

### 4.3. Quantification of Glucosinolate Concentrations

Another set of broccoli plants (three plants for each treatment and each plant considered biological replication) were subjected to the same JA treatments described above for GS analysis. These would provide the herbivory-free control tissue for glucosinolate analysis not influenced by larval feeding. Three days after JA treatments, all broccoli leaves were collected for GS analysis. Above ground aerial leaf and stem samples were frozen in liquid nitrogen, and stored at −20 °C prior to freeze-drying. Freeze-dried tissues were ground into a fine powder using a coffee grinder and stored at −20 °C prior to GS analysis using high-performance liquid chromatography (HPLC, Agilent, Santa Clara, CA, USA). Extraction and quantification of GSs using HPLC was performed using a previously published protocol [34]. Freeze-dried vegetative broccoli plant powder (0.2 g) and 2 mL of 70% methanol were added to 10 mL tubes (Nalgene) and heated on a heating block at 95 °C for 10 min. After cooling on ice, 0.5 mL benzylglucosinolate (100 µM) was added as an internal standard (POS Pilot Plant Corp, Saskatoon, SK, Canada), mixed, and centrifuged at 8000× *g* for 5 min at 4 °C. The supernatant was saved, and the pellet was re-extracted with 2 mL 70% methanol at 95 °C for 10 min; the two extracts were combined. A subsample (1 mL) from each pooled extract was transferred into a 2 mL microcentrifuge tube (Fisher Scientific, Waltham, MA, USA). Protein was precipitated with 0.15 mL of a 1:1 mixture of 1 M lead acetate and 1 M barium acetate. After centrifuging at 12,000× *g* for 1 min, each sample was loaded onto a column containing 1 M NaOH and 1 M pyridine acetate-charged DEAE Sephadex A-25 resin (GE Healthcare, Piscataway, NJ, USA) for desulfation with arylsulfatase (*Helix pomatia* Type-1, Sigma-Aldrich, St. Louis, MO, USA) for 18 h, and the desulfo-GSs were eluted with 3 mL Millipore-filtered ddH_2_O. Samples (100 µL) were injected on to an Agilent 1100 HPLC system (Agilent, Santa Clara, CA, USA), equipped with a G1311A binary pump, a G1322A vacuum degasser, a G1316A thermostatic column compartment, a G1315B diode array detector and an HP 1100 series G1313A autosampler. The UV detector was set at 229 nm wavelength. An all-guard cartridge pre-column (Alltech, Lexington, KY, USA), and a Kromasil RP-C18 column (250 mm × 4.6 mm, 5 µm particle size, Supelco, Bellefonte, PA, USA) were used for quantification. Desulfo-GSs were eluted from the column over 45 min with a linear gradient of 0% to 20% acetonitrile at a flow rate of 1 mL/min. Benzylglucosinolate was used as an internal standard and UV response factors for different types of GSs were used as determined by a previous study [53]. The identification of desulfo-GS profiles were validated by LC-tandem MS using a Waters 32 Q-Tof Ultima spectrometer (Waters, Milford, MA, USA) coupled to a Waters 1525 HPLC system and full scan LC-MS using a Finnigan LCQ Deca XP. The molecular ion and fragmentation patterns of individual desulfo-GSs were matched with the literature for GS identification [54,55].

### 4.4. Quantification of Glucosinolate Hydrolysis Product Concentrations

Freeze-dried broccoli plant tissue (50 mg) was suspended in 1 mL distilled water in a 2 mL microcentrifuge tube (Fisher Scientific, Waltham, MA, USA). Hydrolysis products were generated naturally by endogenous myrosinase in the absence of light at room temperature for 4 h. After adding 1 mL of dichloromethane, the samples were centrifuged at 12,000× *g* for 2 min and the supernatant was collected for analysis using a 6890N gas chromatography (GC) coupled to an 5975B mass spectrometer (MS) detector system equipped with (Agilent Technologies, Santa Clara, CA, USA), an Agilent model 7683B series auto sampler, and a 30 m × 0.32 mm J&W HP-5 capillary column (Agilent Technologies). A 1 μL sample of the dichloromethane extract was injected onto the GC-MS. After an initial temperature hold at 40 °C for 2 min, the oven temperature was increased by 10 °C/min to 260 °C and held for 10 min [56]. The split ratio was 1:1. Injector and detector temperatures were set at 200 and 280 °C, respectively. The flow rate of the helium carrier gas was set at 1.1 mL/min. Ionization mode was set in the electron impact (EI) mode. Ionization source temperature was 250 °C. Full scan mode range was *m*/*z* 40–500. Each GC-MS sample’s results were processed by AMDIS [57] for deconvolution and identification by a user library from authentic standards (see list below) or using the NIST library and the ELU files created as output of AMDIS and saved for identification with the SpectConnect web server [58]. The data were arranged on a three-dimensional matrix consisting of arbitrary peak index (RT-*m/z* pair), sample names (observations), and peak area (variables). Iberin (LKT Laboratories Inc, St Paul, MN, USA), sulforaphane (Sigma-Aldrich, St. Louis, MO, USA), 3-butenyl isothiocyanate (3-butenyl ITC, TCI America, Portland, OR, USA), indole-3-carboxaldehyde (I3CA), 1-methoxyindole-3-carbinol (MI3C), 1-methoxyindole-3-carboxaldehyde (MI3CA), 1-methoxyindole-3-acetonitrile (MI3A) were quantified based on internal standards of phenyl isothiocyanate (Sigma-Aldrich) and expressed as phenyl isothiocyanate equivalent concentrations by adjusting via multiplication with an arbitrary constant value (0.46) to avoid exceeding concentrations of precursors. Peak identification was done by using standard compounds (Iberin, sulforaphane, and 3-butenyl isothiocyanate); otherwise the NIST/EPA/NIH Mass Spectral Library was used [59]. Neoascorbigen and indole-3-carbinol (I3C) were quantitated by HPLC [6].

### 4.5. RNA Extraction and Quantitative Real Time-PCR

Total RNA was isolated individually from each of the three biological replicates (one analytical replication for each biological sample) of control and JA treated broccoli plant tissue samples using the RNeasy Mini Kit (QIAGEN) according to the manufacturer’s instructions. The quantity of RNA was measured using a NanoDrop 3300 spectrophotometer (Thermo Scientific, Waltham, MA, USA). One μg of the total RNA was reverse-transcribed with Superscript™ III First-Strand Synthesis SuperMix for qRT-PCR (Invitrogen, Carlsbad, CA, USA) according to the manufacturer’s instructions. The resulting cDNA samples were diluted to 1/10 their concentrations (*v*/*v*) for qRT-PCR. The primer sets of glucosinolate biosynthesis genes, hydrolysis genes, and transcription factor genes were designed based on database-published sequences [60]. All primers were validated for amplification efficiency and binding specificity by performing qRT-PCR with a serial dilution of bulked broccoli cDNA followed by dissociation curve analysis with the Power SYBR® Green RT-PCR Master Mix (QIAGEN) using an ABI 7900HT Fast Real-Time PCR System (Applied Biosystems, Foster city, CA, USA) according to the manufacturer’s instructions. A final list of the primers used, the gene model from which they were created, and a classification of the gene can be found in Table 1. The qRT-PCR data were expressed after normalization to the broccoli actin gene (*BoACT1*) [61,62] (Figure 3, Figure 4 and Figure S2). The primers were synthesized by Integrated DNA Technologies Inc. (Coralville, IA, USA). Quantitative real-time PCR was carried out with the Power SYBR^®^ Green RT-PCR Master Mix (QIAGEN) using an ABI 7900HT Fast Real-Time PCR System (Applied Biosystems) according to the manufacturer’s instructions. The relative expression ratio was determined with the equation 2^−∆∆*C*t^ using the BoACT1 normalized ∆*C*_t_ values generated by the ABI 7900HT Sequence Detection System Software 2.4 (Applied Biosystems) [6].

### 4.6. Statistical Analysis

One-way ANOVA performed using JMP 12 (SAS, Cary, NC, USA) was used to assess effect of JA on all the insect and plant assays. The significance of differences between treatment means was evaluated with Fisher’s least significant difference (LSD) test at *p* = 0.05 levels. Pearson correlation was conducted on all pairs of data from insect feeding experiments, GS concentrations, GS hydrolysis concentrations, and gene expression based on the mean values of each treatment from two broccoli cultivars.

## Figures and Tables

**Table 1:** Glucosinolate profiles (µM/g DW) of two broccoli cultivars treated with different JA concentrations (µmol/L). Different letters from right side of values indicate differences between treatments within a cultivar for a given glucosinolate according to Fisher’s LSD tests (*p* = 0.05). Abbreviations: GM = Green Magic, GR = glucoraphanin, GBS = glucobrassicin, GN = gluconasturtiin, NeoGBS = neoglucobrassicin.

Cultivar-Treatment	Glucoiberin	GR	Gluconapin	GBS	GN	NeoGBS
GM-0	1.73 ± 0.00 a	1.00 ± 0.02 a	0.85 ± 1.62 a	1.50 ± 0.13 b	1.41 ± 0.42 a	1.07 ± 0.42 a
GM-100	2.26 ± 0.05 b	0.43 ± 0.84 a	1.19 ± 0.18 a	1.05 ± 0.13 a	2.09 ± 1.65 a	4.97 ± 1.65 b
GM-200	2.09 ± 0.13 b	0.67 ± 0.56 a	1.60 ± 0.21 a	1.31 ± 0.18 b	2.30 ± 1.45 a	10.0 ± 1.45 c
GM-400	2.33 ± 0.01 c	0.79 ± 0.27 a	1.70 ± 0.37 a	1.17 ± 0.37 ab	1.85 ± 0.31 a	16.7 ± 0.31 d
VI158-0	0.58 ± 0.00 a	0.24 ± 0.12 a	0.57 ± 0.18 a	0.98 ± 0.00 a	0.53 ± 0.02 d	0.16 ± 0.02 a
VI158-100	1.81 ± 0.00 b	0.24 ± 0.15 a	1.05 ± 0.14 b	3.42 ± 0.07 b	0.18 ± 0.01 b	0.68 ± 0.01 b
VI158-200	0.54 ± 0.00 a	0.76 ± 0.26 b	0.85 ± 0.11 a	5.35 ± 0.09 c	0.26 ± 0.00 c	0.16 ± 0.00 a
VI158-400	0.57 ± 0.00 a	0.32 ± 0.16 a	0.55 ± 0.07 a	7.09 ± 0.05 d	0.13 ± 0.00 a	0.18 ± 0.00 a

**Table 2:** Glucosinolate hydrolysis product profiles (µM/g DW) of two broccoli cultivars treated with different jasmonate (JA) concentrations; Different letters from right side of values indicate differences between treatment/cultivar combinations for a given glucosinolate hydrolysis product according to Fisher’s LSD tests (*p* = 0.05); Abbreviations: GM = Green Magic; ITC = isothiocyanate; I3C = indole-3-carbinol; I3CA = indole-3-carboxaldehyde; MI3C = 1-methoxyindole-3-carbinol; MI3CA = 1-methoxyindole-3-carboxaldehyde; MI3A = 1-methoxyindole-3-acetonitrile; NeoASG = neoascorbigen; SF = sulforaphane.

Cultivar–Treatment	Iberin	SF	3-Butenyl ITC	I3C	I3CA	MI3C	MI3CA	MI3A	NeoASG
GM-0	0.16 ± 0.01 c	1.41 ± 0.10 c	0.23 ± 0.05 b	0.16 ± 0.04 a	0.00 ± 0.00 d	0.53 ± 0.00 d	0.43 ± 0.02 c	0.14 ± 0.02 d	0.38 ± 0.01 c
GM-100	0.35 ± 0.05 a	2.09 ± 0.05 ab	0.40 ± 0.06 a	0.11 ± 0.04 ab	0.24 ± 0.01 c	2.51 ± 0.89 c	1.53 ± 0.08 b	0.30 ± 0.00 d	0.55 ± 0.08 b
GM-200	0.34 ± 0.04 ab	2.30 ± 0.26 a	0.42 ± 0.07 a	0.09 ± 0.02 bc	0.35 ± 0.03 b	3.80 ± 0.65 b	1.68 ± 0.21 b	0.47 ± 0.04 a	0.75 ± 0.10 a
GM-400	0.27 ± 0.04 b	1.85 ± 0.10 b	0.38 ± 0.09 a	0.05 ± 0.01 c	0.92 ± 0.04 a	5.40 ± 0.64 a	2.38 ± 0.13 a	0.39 ± 0.05 b	0.74 ± 0.10 a
VI158-0	0.00 ± 0.00 a	0.53 ± 0.19 a	0.09 ± 0.03 a	0.15 ± 0.06 b	0.81 ± 0.18 a	0.26 ± 0.06 b	0.55 ± 0.21 b	0.00 ± 0.00 a	0.37 ± 0.01 b
VI158-100	0.00 ± 0.00 a	0.18 ± 0.02 b	0.03 ± 0.00 b	0.22 ± 0.02 b	1.99 ± 1.07 a	0.45 ± 0.33 b	0.81 ± 0.48 b	0.00 ± 0.00 a	0.40 ± 0.02 ab
VI158-200	0.00 ± 0.00 a	0.26 ± 0.01 b	0.04 ± 0.01 b	0.35 ± 0.08 a	1.88 ± 0.61 a	0.63 ± 0.44 b	0.83 ± 0.55 b	0.00 ± 0.00 a	0.40 ± 0.04 ab
VI158-400	0.00 ± 0.00 a	0.13 ± 0.01 b	0.03 ± 0.01 b	0.23 ± 0.08 ab	1.60 ± 0.30 a	1.96 ± 0.07 a	1.73 ± 0.22 a	0.02 ± 0.04 a	0.44 ± 0.01 a

## Supplementary Materials

Supplementary materials can be found at http://www.mdpi.com/1422-0067/17/7/1135/s1.

## References

[1] Eberhardt M.V., Kobira K., Keck A.S., Juvik J.A., Jeffery E.H. (2005). Correlation analyses of phytochemical composition, chemical and cellular measures of antioxidant activity of *broccoli* (*Brassica oleracea* L. Var. italica). J. Agric. Food Chem..

[2] Velasco P., Cartea M.E., Gonzalez C., Vilar M., Ordas A. (2007). Factors affecting the glucosinolate content of kale (*Brassica oleracea* acephala group). J. Agric. Food Chem..

[3] Koh E., Wimalasiri K.M.S., Chassy A.W., Mitchell A.E. (2009). Content of ascorbic acid, quercetin, kaempferol and total phenolics in commercial broccoli. J. Food Comp. Anal..

[4] Moon Y.J., Wang X., Morris M.E. (2006). Dietary flavonoids: Effects on xenobiotic and carcinogen metabolism. Toxicol. In Vitro.

[5] Ku K.M., Jeffery E.H., Juvik J.A. (2014). Exogenous methyl jasmonate treatment increases glucosinolate biosynthesis and quinone reductase activity in kale leaf tissue. PLoS ONE.

[6] Ku K.M., Choi J.H., Kim H.S., Kushad M.M., Jeffery E.H., Juvik J.A. (2013). Methyl jasmonate and 1-methylcyclopropene treatment effects on quinone reductase inducing activity and post-harvest quality of broccoli. PLoS ONE.

[7] Ku K.M., Jeffery E.H., Juvik J.A. (2014). Optimization of methyl jasmonate application to broccoli florets to enhance health-promoting phytochemical content. J. Sci. Food Agric..

[8] Ku K.M., Choi J.-H., Kushad M.M., Jeffery E.H., Juvik J.A. (2013). Pre-harvest methyl jasmonate treatment enhances cauliflower chemoprotective attributes without a loss in postharvest quality. Plant Foods Hum. Nutr..

[9] Farmer E.E., Ryan C.A. (1990). Interplant communication-airborne methyl jasmonate induces synthesis of proteinase-inhibitors in plant-leaves. Proc. Natl. Acad. Sci. USA.

[10] Bodnaryk R.P. (1994). Potent effect of jasmonates on indole glucosinolates in oilseed rape and mustard. Phytochemistry.

[11] Baldwin I.T. (1999). Inducible nicotine production in native *nicotiana* as an example of adaptive phenotypic plasticity. J. Chem. Ecol..

[12] Constabel C.P., Bergey D.R., Ryan C.A. (1995). Systemin activates synthesis of wound-inducible tomato leaf polyphenol oxidase via the octadecanoid defense signaling pathway. Proc. Natl. Acad. Sci. USA.

[13] Howe G.A., Jander G. (2008). Plant immunity to insect herbivores. Annu. Rev. Plant Biol..

[14] Thaler J.S., Stout M.J., Karban R., Duffey S.S. (2001). Jasmonate-mediated induced plant resistance affects a community of herbivores. Ecol. Entomol..

[15] Bruinsma M., van Dam N.M., van Loon J.J., Dicke M. (2007). Jasmonic acid-induced changes in Brassica oleracea affect oviposition preference of two specialist herbivores. J. Chem. Ecol..

[16] Beekwilder J., van Leeuwen W., van Dam N.M., Bertossi M., Grandi V., Mizzi L., Soloviev M., Szabados L., Molthoff J.W., Schipper B. (2008). The impact of the absence of aliphatic glucosinolates on insect herbivory in *Arabidopsis*. PLoS ONE.

[17] Mewis I., Appel H.M., Hom A., Raina R., Schultz J.C. (2005). Major signaling pathways modulate *Arabidopsis* glucosinolate accumulation and response to both phloem-feeding and chewing insects. Plant Physiol..

[18] Liang Y.-S., Choi Y.H., Kim H.K., Linthorst H.J.M., Verpoorte R. (2006). Metabolomic analysis of methyl jasmonate treated *Brassica rapa* leaves by 2-dimensional NMR spectroscopy. Phytochemistry.

[19] Ku K.M., Jeffery E.H., Juvik J.A. (2013). Influence of seasonal variation and methyl jasmonate mediated induction of glucosinolate biosynthesis on quinone reductase activity in broccoli florets. J. Agric. Food Chem..

[20] Kim H.S., Juvik J.A. (2011). Effect of selenium fertilization and methyl jasmonate treatment on glucosinolate accumulation in broccoli florets. J. Am. Soc. Horticult. Sci..

[21] Mikkelsen M.D., Hansen C.H., Wittstock U., Halkier B.A. (2000). Cytochrome P450 CYP79B2 from *Arabidopsis* catalyzes the conversion of tryptophan to indole-3-acetaldoxime, a precursor of indole glucosinolates and indole-3-acetic acid. J. Biol. Chem..

[22] Hull A.K., Vij R., Celenza J.L. (2000). *Arabidopsis* cytochrome P450s that catalyze the first step of tryptophan-dependent indole-3-acetic acid biosynthesis. Proc. Natl. Acad. Sci. USA.

[23] Piotrowski M., Schemenewitz A., Lopukhina A., Müller A., Janowitz T., Weiler E.W., Oecking C. (2004). Desulfoglucosinolate sulfotransferases from *Arabidopsis thaliana* catalyze the final step in the biosynthesis of the glucosinolate core structure. J. Biol. Chem..

[24] Pfalz M., Mikkelsen M.D., Bednarek P., Olsen C.E., Halkier B.A., Kroymann J. (2011). Metabolic engineering in nicotiana benthamiana reveals key enzyme functions in *Arabidopsis* indole glucosinolate modification. Plant Cell Online.

[25] Bednarek P., Piślewska-Bednarek M., Svatoš A., Schneider B., Doubský J., Mansurova M., Humphry M., Consonni C., Panstruga R., Sanchez-Vallet A. (2009). A glucosinolate metabolism pathway in living plant cells mediates broad-spectrum antifungal defense. Science.

[26] Kim J.H., Jander G. (2007). *Myzus persicae* (green peach aphid) feeding on Arabidopsis induces the formation of a deterrent indole glucosinolate. Plant J..

[27] Burow M., Zhang Z.-Y., Ober J.A., Lambrix V.M., Wittstock U., Gershenzon J., Kliebenstein D.J. (2008). ESP and ESM1 mediate indol-3-acetonitrile production from indol-3-ylmethyl glucosinolate in *Arabidopsis*. Phytochemistry.

[28] Baasanjav-Gerber C., Monien B.H., Mewis I., Schreiner M., Barillari J., Iori R., Glatt H. (2011). Identification of glucosinolate congeners able to form DNA adducts and to induce mutations upon activation by myrosinase. Mol. Nutr. Food Res..

[29] Glatt H., Baasanjav-Gerber C., Schumacher F., Monien B.H., Schreiner M., Frank H., Seidel A., Engst W. (2011). 1-Methoxy-3-indolylmethyl glucosinolate; a potent genotoxicant in bacterial and mammalian cells: Mechanisms of bioactivation. Chem.-Biol. Interact..

[30] Renwick J.A.A., Chew F.S. (1994). Oviposition behavior in lepidoptera. Annu. Rev. Entomol..

[31] Müller R., Vos M., Sun J.Y., Sønderby I.E., Halkier B.A., Wittstock U., Jander G. (2010). Differential effects of indole and aliphatic glucosinolates on lepidopteran herbivores. J. Chem. Ecol..

[32] Kim J.H., Lee B.W., Schroeder F.C., Jander G. (2008). Identification of indole glucosinolate breakdown products with antifeedant effects on *Myzus persicae* (green peach aphid). Plant J..

[33] Kliebenstein D.J., Kroymann J., Brown P., Figuth A., Pedersen D., Gershenzon J., Mitchell-Olds T. (2001). Genetic control of natural variation in *Arabidopsis* glucosinolate accumulation. Plant Physiol..

[34] Brown A.F., Yousef G.G., Jeffery E.H., Klein B.P., Wallig M.A., Kushad M.M., Juvik J.A. (2002). Glucosinolate profiles in broccoli: Variation in levels and implications in breeding for cancer chemoprotection. J. Am. Soc. Horticult. Sci..

[35] Scriber J.M., Slansky F. (1981). The nutritional ecology of immature insects. Annu. Rev. Entomol..

[36] Lincoln D.E., Newton T.S., Ehrlich P.R., Williams K.S. (1982). Coevolution of the checkerspot butterfly *Euphydryas chalcedona* and its larval food plant *Diplacus aurantiacus*: Larval response to protein and leaf resin. Oecologia.

[37] Zalucki M.P., Clarke A.R., Malcolm S.B. (2002). Ecology and behavior of first instar larval *Lepidoptera*. Annu. Rev. Entomol..

[38] Frerigmann H., Gigolashvili T. (2014). MYB34, MYB51, and MYB122 distinctly regulate indolic glucosinolate biosynthesis in *Arabidopsis thaliana*. Mol. Plant.

[39] Agerbirk N., Olsen C.E., Sørensen H. (1998). Initial and final products, nitriles, and ascorbigens produced in myrosinase-catalyzed hydrolysis of indole glucosinolates. J. Agric. Food Chem..

[40] Matusheski N.V., Swarup R., Juvik J.A., Mithen R., Bennett M., Jeffery E.H. (2006). Epithiospecifier protein from broccoli (*Brassica oleracea* L. *ssp. italica*) inhibits formation of the anticancer agent sulforaphane. J. Agric. Food Chem..

[41] Kong X.Y., Kissen R., Bones A.M. (2012). Characterization of recombinant nitrile-specifier proteins (NSPs) of Arabidopsis thaliana: Dependency on Fe(II) ions and the effect of glucosinolate substrate and reaction conditions. Phytochemistry.

[42] Van Dam N.M., Oomen M.W. (2008). Root and shoot jasmonic acid applications differentially affect leaf chemistry and herbivore growth. Plant Signal. Behav..

[43] Harvey J.A., Gols R., Wagenaar R., Bezemer T.M. (2007). Development of an insect herbivore and its pupal parasitoid reflect differences in direct plant defense. J. Chem. Ecol..

[44] Harvey J., Dam N., Raaijmakers C., Bullock J., Gols R. (2011). Tri-trophic effects of inter- and intra-population variation in defence chemistry of wild cabbage (*Brassica oleracea*). Oecologia.

[45] Stephensen P.U., Bonnesen C., Schaldach C., Andersen O., Bjeldanes L.F., Vang O. (2000). *N*-methoxyindole-3-carbinol is a more efficient inducer of cytochrome P-450 1A1 in cultured cells than indol-3-carbinol. Nutr. Cancer.

[46] Neave A.S., Sarup S.M., Seidelin M., Duus F., Vang O. (2005). Characterization of the *N*-methoxyindole-3-carbinol (NI3C), induced cell cycle arrest in human colon cancer cell lines. Toxicol. Sci..

[47] Haack M., Lowinger M., Lippmann D., Kipp A., Pagnotta E., Iori R., Monien B.H., Glatt H., Brauer M.N., Wessjohann L.A. (2010). Breakdown products of neoglucobrassicin inhibit activation of Nrf2 target genes mediated by myrosinase-derived glucoraphanin hydrolysis products. Biol. Chem..

[48] Das N., Berhow M., Jeffery E. (2014). Comparison of bioactivity between sulforaphane and neoglucobrassicin hydrolysis product in murine and human cell lines (372.5). FASEB J..

[49] West L.G., Windsor N.L., Gaonkar A.G., Matusheski N.V., Kim N., Ludwig C.J., Lawrence L.L. (2008). Enteric-Coated Glucosinolates and β-Thioglucosidases.

[50] Wiesner M., Hanschen F.S., Schreiner M., Glatt H., Zrenner R. (2013). Induced production of 1-methoxy-indol-3-ylmethyl glucosinolate by jasmonic acid and methyl jasmonate in sprouts and leaves of pak choi (*Brassica rapa* ssp. chinensis). Int. J. Mol. Sci..

[51] Chen Y.Z., Pang Q.Y., He Y., Zhu N., Branstrom I., Yan X.F., Chen S. (2012). Proteomics and metabolomics of *Arabidopsis* responses to perturbation of glucosinolate biosynthesis. Mol. Plant.

[52] Huot B., Yao J., Montgomery B.L., He S.Y. (2014). Growth-defense tradeoffs in plants: A balancing act to optimize fitness. Mol. Plant.

[53] Wathelet J.P., Marlier M., Severin M., Boenke A., Wagstaffe P.J. (1995). Measurement of glucosinolates in rapeseeds. Nat. Toxins.

[54] Tian Q., Rosselot R.A., Schwartz S.J. (2005). Quantitative determination of intact glucosinolates in broccoli, broccoli sprouts, Brussels sprouts, and cauliflower by high-performance liquid chromatography-electrospray ionization-tandem mass spectrometry. Anal. Biochem..

[55] Velasco P., Francisco M., Moreno D.A., Ferreres F., García-Viguera C., Cartea M.E. (2011). Phytochemical fingerprinting of vegetable *Brassica oleracea* and *Brassica napus* by simultaneous identification of glucosinolates and phenolics. Phytochem. Anal..

[56] Dosz E.B., Ku K.M., Juvik J.A., Jeffery E.H. (2014). Total myrosinase activity estimates in brassica vegetable produce. J. Agric. Food Chem..

[57] Mallard M.G., Reed J. Amdis–User Guide. http://chemdata.nist.gov/mass-spc/amdis/docs/amdis.pdf.

[58] Styczynski M.P., Moxley J.F., Tong L.V., Walther J.L., Jensen K.L., Stephanopoulos G.N. (2007). Systematic identification of conserved metabolites in GC/MS data for metabolomics and biomarker discovery. Anal. Chem..

[59] National Institute of Standards and Technology. http://www.nist.gov/srd/nist1a.cfm.

[60] Liu S., Liu Y., Yang X., Tong C., Edwards D., Parkin I.A., Zhao M., Ma J., Yu J., Huang S. (2014). The *Brassica oleracea* genome reveals the asymmetrical evolution of polyploid genomes. Nat. Commun..

[61] Kim H.S. (2011). Functional Studies of Lignin Biosynthesis Genes and Putative Flowering Gene in Miscanthus x Giganteus and Studies on Indolyl Glucosinolate Biosynthesis and Translocation in *Brassica oleracea*. Ph. D. Thesis.

[62] Hasperué J.H., Gómez-Lobato M.E., Chaves A.R., Civello P.M., Martínez G.A. (2013). Time of day at harvest affects the expression of chlorophyll degrading genes during postharvest storage of broccoli. Postharvest Biol. Technol..

