# Supplementary material for: Transcriptome and Metabolome Analyses of Glucosinolates in Two Broccoli Cultivars Following Jasmonate Treatment for the Induction of Glucosinolate Defense to Trichoplusia ni (Hübner)
Source: Int J Mol Sci. 2016 Jul 15;17(7):1135. doi: 10.3390/ijms17071135 (PMC4964508; doi:10.3390/ijms17071135)
Supplement: Supplementary file 1 [file ijms-17-01135-s001.pdf]

Supplementary Materials: Transcriptome and Metabolome Analyses of Glucosinolates in Two Broccoli Cultivars Following Jasmonate Treatment for the Induction of Glucosinolate Defense to *Trichoplusia ni* (Hübner)

Kang-Mo Ku, Talon M. Becker and John A. Juvik

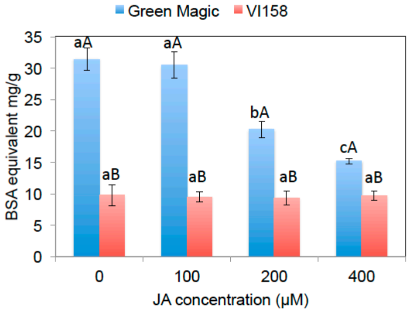

Figure S1. Crude protein estimates reported as bovine serum albumin (BSA) equivalents (mg/g DW). Different lowercase letters indicate differences between treatments within a variety and uppercase letters indicate differences between accessions within a treatment according to Fisher's LSD tests ($p = 0.05$).

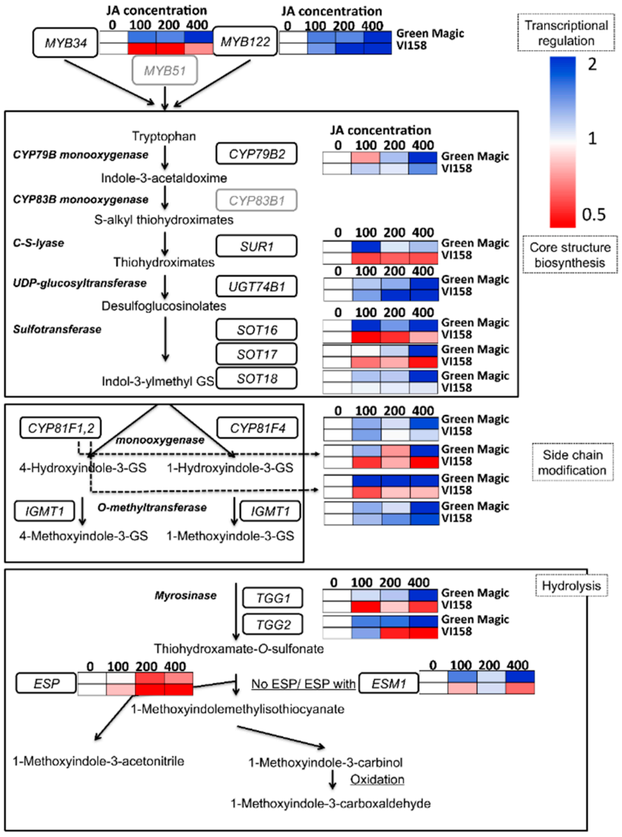

Figure S2. Glucosinolates related genes expression 3 days after JA treatments. The color scale represents log2-transformed, actin normalized (*BoACT1*) gene expression ratios comparing transcript abundance of JA treated plants following 100, 200, and 400 μM JA treatments to control, uninfested plants.

Table S1. Primer set information for gene expression measurement.

Gene Class	Gene Name	Gene Model	Type	Sequence
Indole-Associated Transcription Factors	MYB34	Bol017062	Forward	TGAAGGTGGATGGCGTACTCT
			Reverse	GCCCATCTCAGCCTACAACTCT
	MYB122	Bol026204	Forward	CTTCCCGACAAAGCTGGACT
			Reverse	TTGGCTAAACTCACCACGCT
Core Structure Biosynthesis Genes	CYP79B2	Bol032767	Forward	GATGAAATTAAACCCACCATTAAGGA
			Reverse	GCCATGGCCCATTCGA
	SUR1	Bol029775	Forward	GCTCCCACGTCCCGTTT
			Reverse	GCGAACCTCGAGACCACTGT
	UGT74B1	Bol005786	Forward	CGACGGCCACGACTTCAT
			Reverse	GCTTGAAGGATTCGGAGTATGC
	SOT16	Bol039395	Forward	TTCGACGACGCCACGAA
			Reverse	CTCCACGTAAGGCACGAACTC
	SOT17	Bol030757	Forward	CCATCGCCACGCTTCCT
			Reverse	CCGCCGTACTCGACGAAA
	SOT18	Bol026202	Forward	CCCAAAGACAGGCACCACTT
			Reverse	GGAATCGTCGAAGCGAGATC
Indole Side-Chain Modification Genes	CYP81F1	Bol028913	Forward	CCGAGACATTCCGGCTATTC
			Reverse	CATGTCCTCCGTCGGTCTTC
	CYP81F2	Bol026044	Forward	TCTCCCACCAGGACCAACTC
			Reverse	GGTGGACCGGCGGTTT
	IGMT1	Bol007030	Forward	GGACCGGATGCTTCGTCTAC
			Reverse	TCTCTCGCCCTTTCCAAACTT
	CYP81F4	Bol032712	Forward	TCCCTCTCCGCCTCACTCT
			Reverse	GGTGGACGGGAGGTTTAATGA
Myrosinases	TGG1	Bol017328	Forward	GTGCCTACGAGAGGCTATTCAAC
			Reverse	GCCGTAACATCTTTCATCAACCT
	TGG2	Bol028319	Forward	CGAACTCAACGCTACTGGTTACA
			Reverse	TACTCCCCTGCTCCTCTTTCC
Specifier Proteins	ESP	Bol006378	Forward	CTACACGACTGCTACCGTCTATGG
			Reverse	GGTTGTTGGTGGGACGTTTT
	ESM1	Bol005067	Forward	TCCGATGTTGAACCAGTTTGC
			Reverse	CGAAGGATGGCGTTGTAGAAA
Endogenous Control Gene	Actin	Bol030974	Forward	TCCCGAGAGGAAGTACAGTGTCT
			Reverse	GAGATCCACATCTGCTGGAATG

Table S2. Correlations between glucosinolates, their hydrolysis products, and cabbage looper growth parameters. Pearson correlation coefficients were calculated based on mean values of variables for the two different broccoli cultivars.

Variable	By Variable	Correlation	*p*-Value
Caterpillar weight at 5th day	Indole-3-carboxaldehyde	−0.722	0.043
Caterpillar weight at 5th day	Protein concentration	0.840	0.009
% survival	1-Methoxyindole-3-carboxaldehyde	−0.728	0.041
% survival	Total indole GS	−0.810	0.015
% survival	Caterpillar weight at 5th day	0.760	0.029
% survival	Number of days to pupation	−0.819	0.013
Pupal weight	Protein concentration	0.917	0.001
Pupal weight	Caterpillar weight at 5th day	0.806	0.016
Number of days to pupation	1-Methoxyindole-3-carboxaldehyde	0.929	<0.0001
Number of days to pupation	1-Methoxyindole-3-carbinol	0.927	<0.0001
Number of days to pupation	Neoascorbigen	0.860	0.006
Number of days to pupation	Total indole GS	0.977	<0.0001
Number of days to pupation	Total GS	0.913	0.002
